# Supplementary material for: Contextual fear memory retrieval by correlated ensembles of ventral CA1 neurons
Source: Nat Commun. 2020 Jul 13;11:3492. doi: 10.1038/s41467-020-17270-w (PMC7359370; doi:10.1038/s41467-020-17270-w)
Supplement: Supplementary file 1 — Supplementary Information [file 41467_2020_17270_MOESM1_ESM.pdf]

Contextual fear memory retrieval by correlated ensembles of ventral CA1 neurons

Jimenez *et al.*

Supplementary Information

Supplementary Figure 1. The vCA1-BA projection has more reliable shock responses than the vCA1-LHA projection

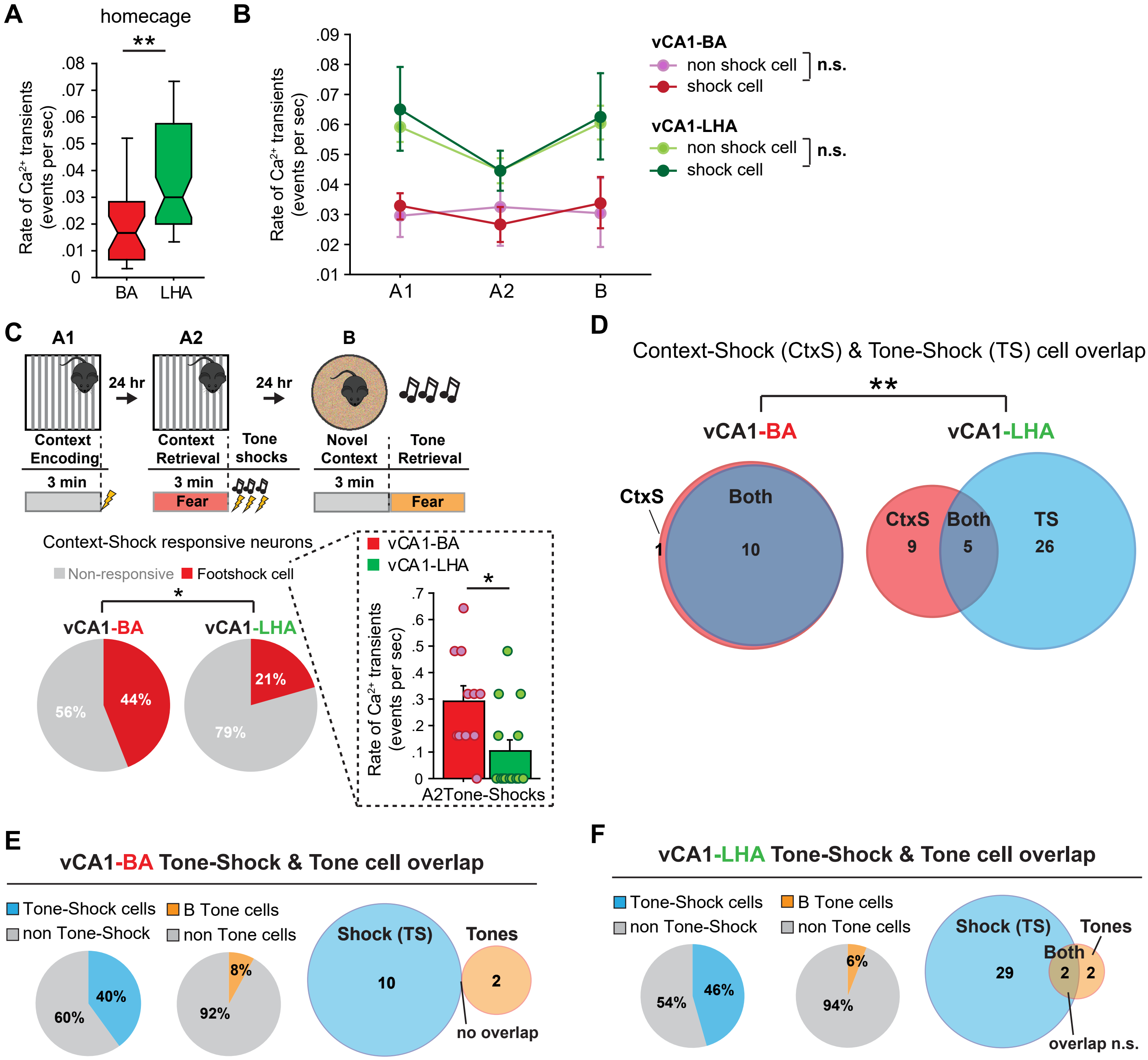

Supplementary Figure 1. The vCA1-BA projection has more reliable shock responses than the vCA1-LHA projection
a, The vCA1-LHA projection has a higher rate of Ca^{2+} transients in the homecage relative to the vCA1-BA projection (Mann Whitney N_{BA}=29, N_{LHA}=61, Z=-3.51, **p=0.0005). **b,** vCA1-BA and LHA shock cells have similar Ca^{2+} transient rates across context conditioning days as non shock cells (Mann-Whitney shock vs non shock cells | BA N_{shock}=11 $N_{nonshock}$=14; A1: U=54.50 p=0.22, A2: U=64.50 p=0.49, B: U=62.50 p=0.43 | LHA N_{shock}=14, $N_{nonshock}$=54; A1: U=377.00 p=0.99, A2: U=375.00 p=0.96, B: U=354.50 p=0.72). **c,** Top: Experimental design, vCA1 was imaged over 3 days while mice explored contexts A1, A2, and B for 3 minutes. Mice received a 2-second foot shock at the end of A1, and three 20-second tones that were paired with a 2-second shock at the end of A2. After 3 minutes in neutral context B, mice were exposed to the same three tones again. Bottom: vCA1-BA shock responsive neurons from A1 encoding have a higher rate of Ca^{2+} activity during exposure to tone shocks relative to vCA1-LHA projecting neurons (Mann Whitney N_{BA}=11, N_{LHA}=14, Z=-2.44, *p=0.0148). **d,** Most vCA1-BA context-shock responsive neurons are also tone-shock responsive neurons while vCA1-LHA shock responsive neurons across shock exposures are significantly less overlapping (Chi squared test of proportions $X^2(2)$=25.98, **p<0.0001 N_{BA}=11, N_{LHA}=40). **e,** vCA1-BA tone-shock cells (from A2, blue in left pie chart and right venn diagram) do not respond to tones in B (yellow in right pie chart and right venn diagram) ($N_{tone\text{-}shock}$=10, N_{tones}=2; random sample overlap control analyses, 2SD range of cell overlap from mock distribution upper= 108.50% lower=26.99%; true overlap=0% Z=1.20, p=0.23). **f,** vCA1-LHA tone-shock cells (from A2, blue in left pie chart and right venn diagram) do not respond to tones in B (yellow in right pie chart and right venn diagram) ($N_{tone\text{-}shock}$=31, N_{tones}=4; random sample overlap control analyses, 2SD range of cell overlap from mock distribution upper= 94.17% lower=3.19%; true overlap=50% Z=0.19, p=0.85).
Error bars, +/- s.e.m. Box plots, min/max, interquartile range, median. Statistical tests comparing distributions were two-sided. Source data are provided as a Source Data file.

Supplementary Figure 2. Imaging vCA1 correlated activity during contextual fear conditioning

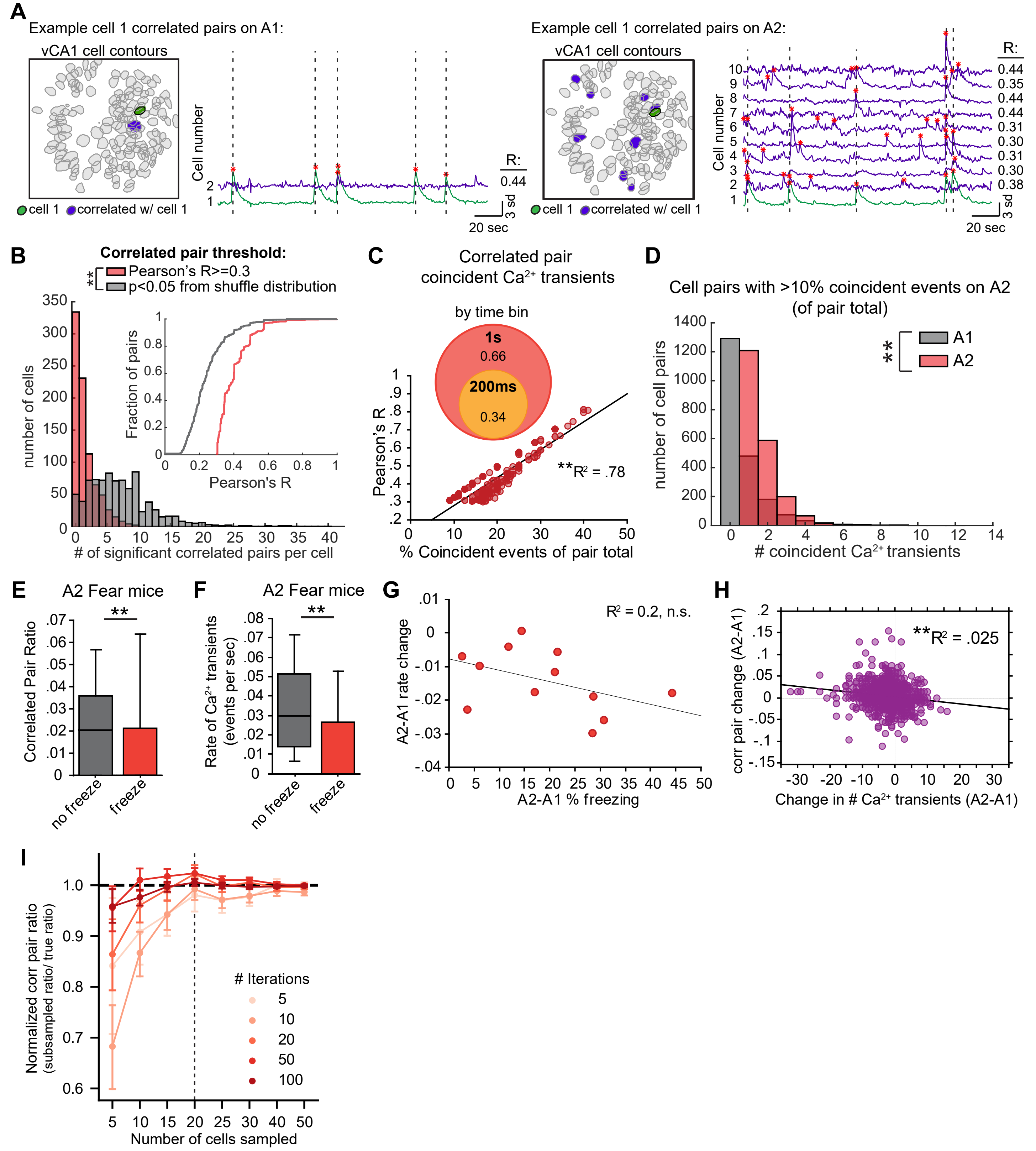

Supplementary Figure 2. Imaging vCA1 correlated activity during contextual fear conditioning
a, Example Ca^{2+} traces of vCA1 correlated pairs during context encoding (left) and retrieval (right). The same cell is tracked across days (cell 1, green in FOV contours plot and Ca^{2+} trace, with cell pairs on each day labeled in purple). **b,** Pearson's R >=0.3 (pink histogram and line in CDF plot) is a higher threshold and more stringent criteria for defining correlated pairs than R thresholds defined by p<0.05 from bootstrap distributions, as ~40% of significant R thresholds from shuffle were <0.2 (inset, CDF plot, gray line), and resulted in more correlated pairs per cell (histogram, gray bars; KStest; N_{cells}=848, KS stat= 0.610, **$p<0.0001$). **c,** % coincident Ca^{2+} events of pair total is correlated with Pearson's R threshold (linear regression $F_{(1,1130)}$=3911.64, R^2=0.78, **$p<0.0001$). Inset: 34% of coincident Ca^{2+} events from correlated pairs defined by activity within 1 sec time bins (pink circle) are also coincident within 200 ms time bins (yellow circle). **d,** Cell pairs with >10% coincident events during A2 retrieval (pink) have more coincident Ca^{2+} transients in A2 than they had during A1

Supplementary Figure 2. Imaging vCA1 correlated activity during contextual fear conditioning (continued)
encoding (gray) (wilcoxon sign rank A2-A1, N_{pairs}=2091, Z=-28.38, p<0.0001). **e,** vCA1 neurons have more correlated pairs during non-freezing bouts than freezing bouts during A2 retrieval (wilcoxon sign rank, N_{cells}=848, Z=-7.715, p<0.0001). **f,** vCA1 Ca^{2+} rate is significantly lower during freezing bouts than non-freezing bouts during A2 (wilcoxon sign rank, N_{cells}=848, Z=-13,70, p<0.0001). **g,** The Ca^{2+} rate change during context retrieval is not correlated with % time freezing in vCA1 Fear mice (linear regression; N_{mice}=12, $F_{(1,10)}$=2.50, R^2=0.2, p=0.14). **h,** The change in vCA1 Ca^{2+} activity during context retrieval is not strongly correlated with the change in correlated pairs (linear regression, N_{cells}=848, $F_{(1,846)}$=22.10, R^2=0.025, p<0.0001). **i,** The minimum # of cells in an FOV needed to estimate the true correlated activity within a vCA1 cell population was calculated by subsampling different numbers of cells within an FOV (x-axis) and calculating a normalized corr pair ratio (subsampled corr pair ratio/ true ratio; y axis). This normalized corr pair ratio was compared across multiple subsampling iterations, and a 20 cell cut off (dotted line, minimum number of cells sampled for normalized corr pair ratio to approach 1.0 for all iterations) was extrapolated as the minimum number of cells needed in an FOV for an imaging mouse to be included in correlated activity analysis throughout the manuscript. All FOVs with >=50 cells were included in this analysis (N_{mice}=9, N_{cells}=847).
Error bars, +/- s.e.m. Box plots, min/max, interquartile range, median. Statistical tests comparing distributions were two-sided. Source data are provided as a Source Data file.

Supplementary Figure 3. vCA1 neurons exhibit increased correlated activity during fear memory retrieval

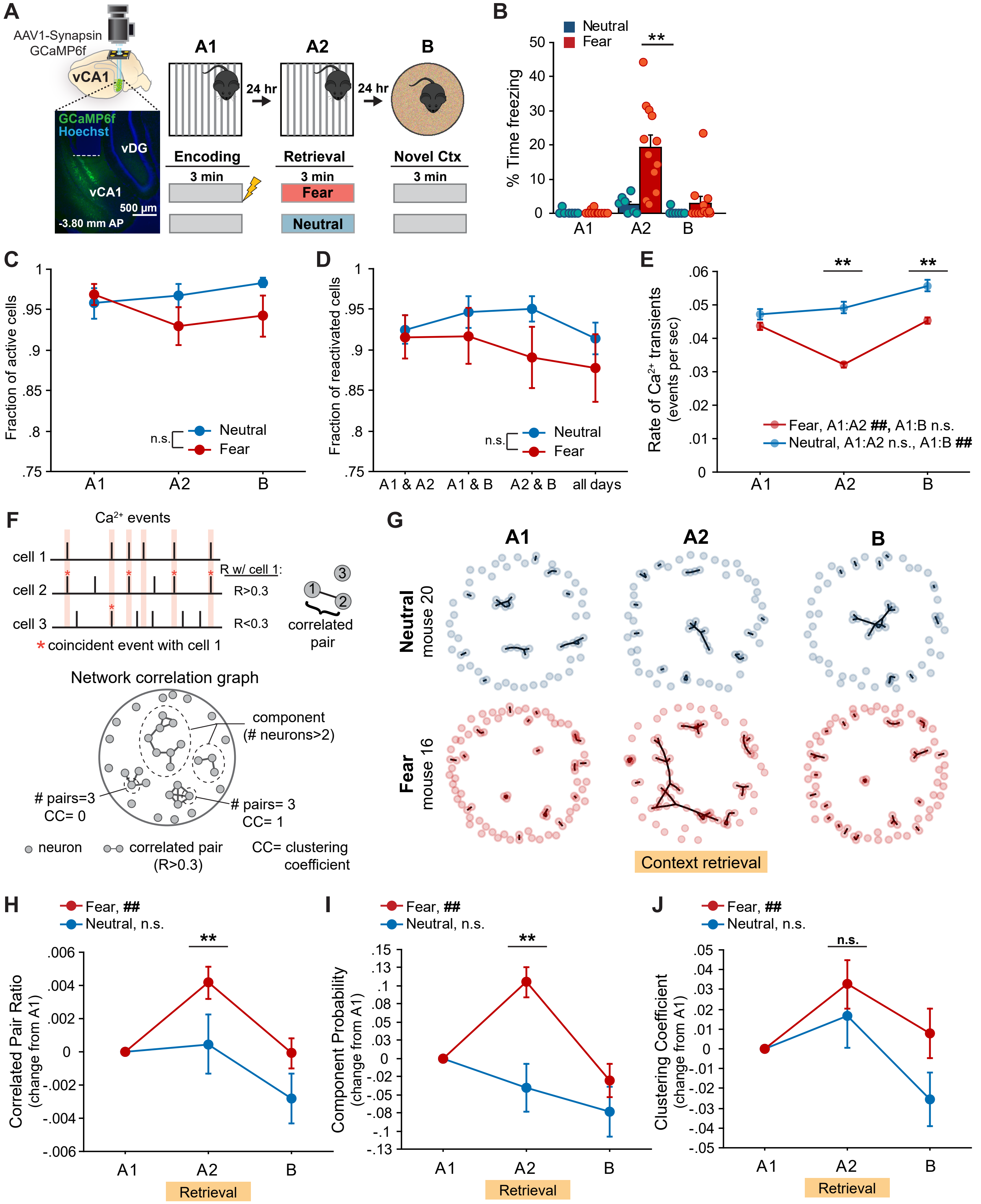

Supplementary Figure 3. vCA1 neurons exhibit increased correlated activity during fear memory retrieval

a, Experimental design for vCA1 Ca^{2+} imaging in contextual conditioning. GCaMP6f was virally expressed and a GRIN lens implanted to target the vCA1 pyramidal layer (left). Ca^{2+} activity was imaged across days; Fear mice (but not Neutral) received a foot shock at the end of A1 (right). The main effect of this experiment was replicated in an independent dataset within the lab. **b,** Contextual conditioning elicits freezing behavior during A2 in Fear mice, but not Neutral (repeated-measures ANOVA; % freezing*group interaction, $F_{(1,17)}$=11.38, **p=0.0010, $N_{Neutral}$=7, N_{Fear}=12). **c,** The fraction of vCA1 active cells/FOV across conditioning days is similar between Neutral and Fear mice ($N_{Neutral}$=7, N_{Fear}=12; Mann-Whitney between groups with bonferroni alpha correction; A1: U=36.00 p=0.61, A2: U=30.00 p=0.31, B: U=30.50 p=0.33). **d,** The fraction of re-activated vCA1 cells across context conditioning days is similar between Neutral and Fear mice ($N_{Neutral}$=7, N_{Fear}=12; Mann-Whitney between groups with bonferroni alpha correction; A1&A2: U=39.00 p=0.80, A1&B: U=39.50 p=0.83, A2&B: U=26.50 p=0.19, all days: U=40.00 p=0.87). **e,** vCA1 neurons exhibit a significant decrease in Ca^{2+} transient rate during A2 in Fear mice but not Neutral (Fear N_{cells}=848, Neutral N_{cells}=367; Mann-Whitney between groups with bonferroni alpha correction A1 p=0.0430 U=144245.00, A2 **p<0.0001 U=106403.00, B **p<0.0001 U=128557.50 | wilcoxon sign rank with bonferroni alpha correction; A1,A2; Fear Z=-10.872 ##p<0.0001, Neutral Z=-1.52 p=0.13; B,A1 Fear Z=-1.56 p=0.12, Neutral Z=4.30, #p<0.0001). **f,** Network correlation graph analysis design; Top: mock traces of Ca^{2+} events demonstrating calculation of significantly correlated pairs (Pearson's R>0.3, cell 1 correlated with cell 2, but not cell 3). Bottom: Example of a network correlation graph with extrapolated graph parameters constructed using Pearson's R correlation matrix. Cell pairs with significant correlation coefficient are connected by a line. **g,** Example correlation graphs across conditioning days in a Neutral mouse (top) and Fear mouse (bottom). **h-j,** (Fear N_{cells}=848, Neutral N_{cells}=367; Mann-Whitney between groups with bonferroni alpha correction; corr pair ratio: A2-A1 **p=0.0083 U=140775.50, B-A1 p=0.12 U=146792.50; comp memb: A2-A1 **p=0.0013 U=137495.50, B-A1 p=0.35 U=150368.00; CC: A2-A1 p=0.10 U=146279.50, B-A1 p=0.37 U=150603.50 | wilcoxon sign rank, A1-A2; corr pair ratio: Fear Z=-4.28, ##p<0.0001, Neutral Z=-0.80, p=0.43; comp probability Fear Z=-4.20, ##p<0.0001, Neutral Z=-1.07, p=0.27; CC Fear Z=-2.86, ##p=0.0042, Neutral Z=-0.939 p=0.35;)

Error bars, +/- s.e.m. Statistical tests comparing distributions were two-sided. Source data are provided as a Source Data file.

Supplementary Figure 4. vCA1 shock cell correlated activity during memory retrieval

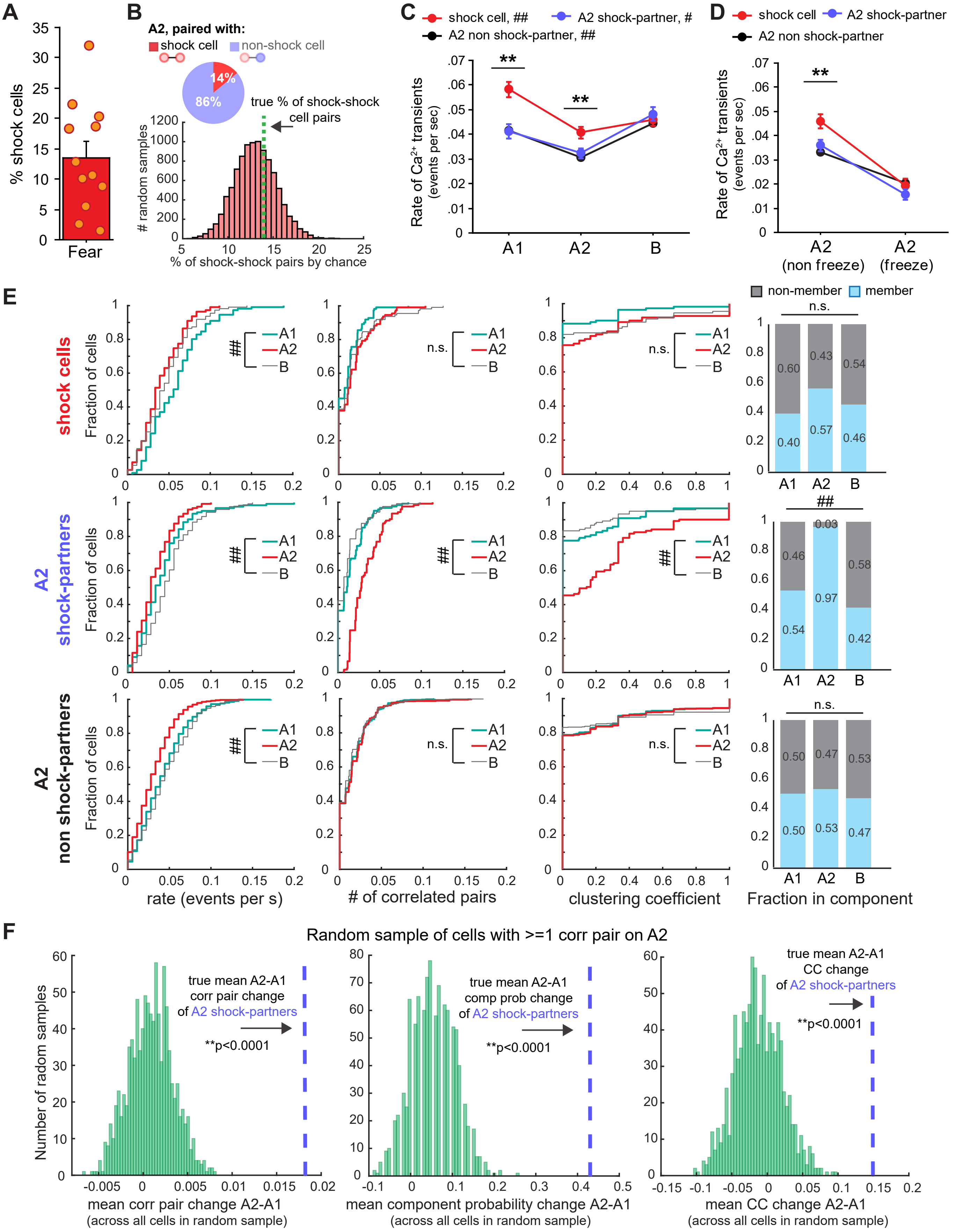

Supplementary Figure 4. vCA1 shock cell correlated activity during memory retrieval

a, Distribution of % of vCA1 shock cells across Fear mice FOVs (N_{mice}=12). **b,** vCA1 shock cells are correlated with other shock cells (left, red slice of pie chart) at chance levels when compared to shuffle distribution (right, histogram; Z-test; true % of shock-shock (14%) to random sample distribution, p=0.71, Z=0.37). **c,** Rate of vCA1 subpopulation Ca^{2+} activity across context conditioning days ((N_{shock}=111, $N_{A2\ shock\text{-}partner}$=121, $N_{A2\ non\ shock\text{-}partner}$=616; Kruskal-Wallis between groups with bonferroni corrected alpha: A1 **p<0.0001 H(2)=26.90, A2 **p<0.0001 H(2)=17.33, B p=0.48 H(2)=1.48; wilcoxon sign rank A1,A2; shock: N_{cells}=111, ##p<0.0001, Z=-5.38; A2 shock-partner: N_{cells}=121, #p=0.0124, Z=-2.50; A2 non shock-partner: N_{cells}=616, ##p<0.0001, Z=-9.30). **d,** A2 Ca^{2+} activity rate during non-freeze and freeze bouts of vCA1 subpopulations (Kruskal-Wallis between groups with bonferroni corrected alpha: non-freeze **p<0.0001 H(2)=20.04, freeze p=0.99 H(2)=0.03). **e,** Population distribution of vCA1 subpopulation Ca^{2+} rates (left column), correlated pair ratio, clustering coefficient, and fraction in component (right column) across context conditioning days (Friedman A1,A2,B; rate: shock ##p<0.0001 $X^2(2)$=33.75, A2 shock-partner ##p<0.0001 $X^2(2)$=20.62, A2 non shock-partner ##p<0.0001 $X^2(2)$=121.31; corr pair ratio: shock p=0.0810 $X^2(2)$=5.03, A2 shock-partner ##p<0.0001 $X^2(2)$=72.41, A2 non shock-partner p=0.26 $X^2(2)$=2.73; CC: shock p=0.44 $X^2(2)$=1.63, A2 shock-partner ##p<0.0001 $X^2(2)$=25.84, A2 non shock-partner p=0.54 $X^2(2)$=1.22; fraction in component: shock p=0.15 $X^2(2)$=3.74, A2 shock-partner ##p<0.0001 $X^2(2)$=44.98, A2 non shock-partner p=0.27 $X^2(2)$=2.50). **f,** Control for A2 shock-partner selection bias (defined by having at least one correlated pair on A2); A matching number of non-shock cells with at least 1 correlated pair in A2 were randomly selected and mean correlation graph parameters were used to create a shuffle distribution for change A2-A1 in # correlated pairs (left), component probability (middle) and CC changes (right). The true mean change in A2-A1 correlation graph parameters in the A2 shock-partner population was significantly greater than these shuffle distributions (Z-test; true mean change A2-A1 to mean change of random sample distribution; # corr pair: p<0.0001, Z=7.05; comp memb: p<0.0001, Z=7.15; CC: p<0.0001, Z=4.94).

Error bars, +/- s.e.m. Statistical tests comparing distributions were two-sided. Source data are provided as a Source Data file.

Supplementary Figure 5. Spatial distribution and reorganization of vCA1 correlated cell pairs

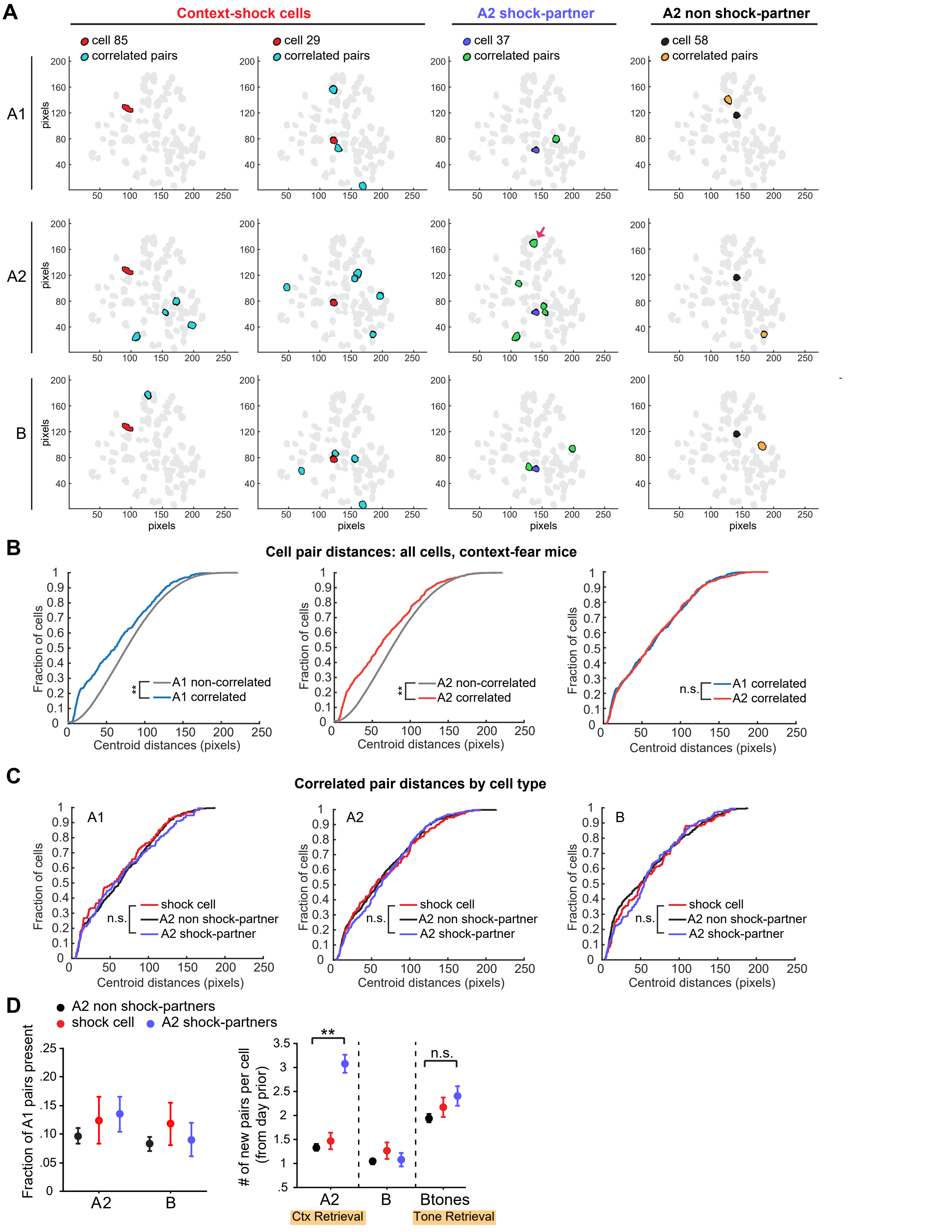

Supplementary Figure 5. Spatial distribution and reorganization of vCA1 correlated cell pairs

a, Example vCA1 spatial contours of an index cell and its correlated cell pairs across days (A1 top, A2 middle, B bottom panels). Example index cells were selected for each cell type (context-shock cell: left 2 columns, red cells; A2 shock-partner cell: 3rd column from the left, blue cell; A2 non shock-partner: right column, black cell). The red arrow on A2 for the A2 shock-partner cell map indicates the spatial contour of the context-shock cell with which it is correlated. **b,** The centroid distances between vCA1 correlated cell pairs are significantly smaller than distances between non-correlated cell pairs in A1 (left, KStest; KS stat= 0.193 **p<0.0001) and A2 (middle, KStest; KS stat= 0.191 **p<0.0001), but correlated cell pairs have similar distances on A1 and A2 (right, KStest; KS stat= 0.040 p=0.21);(N_{cells}=848 for all). **c,** Correlated pair cell distances are similar between vCA1 cell types across all context conditioning days (Kruskal-Wallis correlated pair distances for shock, A2 shock-partner, and A2 non shock-partner; A1: correlated pairs per population N_{shock}=123, $N_{partner}$=212, $N_{nonpartner}$=968, H(2)=1.13, p=0.57; A2: correlated pairs per population N_{shock}=378, $N_{partner}$=406, $N_{nonpartner}$=944, H(2)=1.47, p=0.48; B: correlated pairs per population N_{shock}=135, $N_{partner}$=198, $N_{nonpartner}$=668, H(2)=1.75, p=0.42). **d,** Left; A minority of correlated pairs on A1 persist in A2 and B, and does not vary by vCA1 cell-type (Kruskal-Wallis by cell type, N_{shock}=111, $N_{A2\ shock\text{-}partner}$=121, $N_{A2\ non\ shock\text{-}partner}$=616; A2: H(2)=3.38 p=0.18, B: H(2)=0.18 p=0.92). Right; A2 shock-partner cells are specialized to exhibit an increased number of correlated pairs during context retrieval A2 (left), but not to novel context B (middle), or during B tones retrieval (right) (Kruskal-Wallis by cell type, N_{shock}=111, $N_{A2\ shock\text{-}partner}$=121, $N_{A2\ non\ shock\text{-}partner}$=616; A2: H(2)=97.46 **p<0.0001, B: H(2)=0.83 p=0.66; Btones: H(2)=4.79 p=0.09). Error bars, +/- s.e.m. Statistical tests comparing distributions were two-sided. Source data are provided as a Source Data file.

Supplementary Figure 6. Increased vCA1 correlated activity during memory retrieval is not driven by cell-type rate or R threshold differences

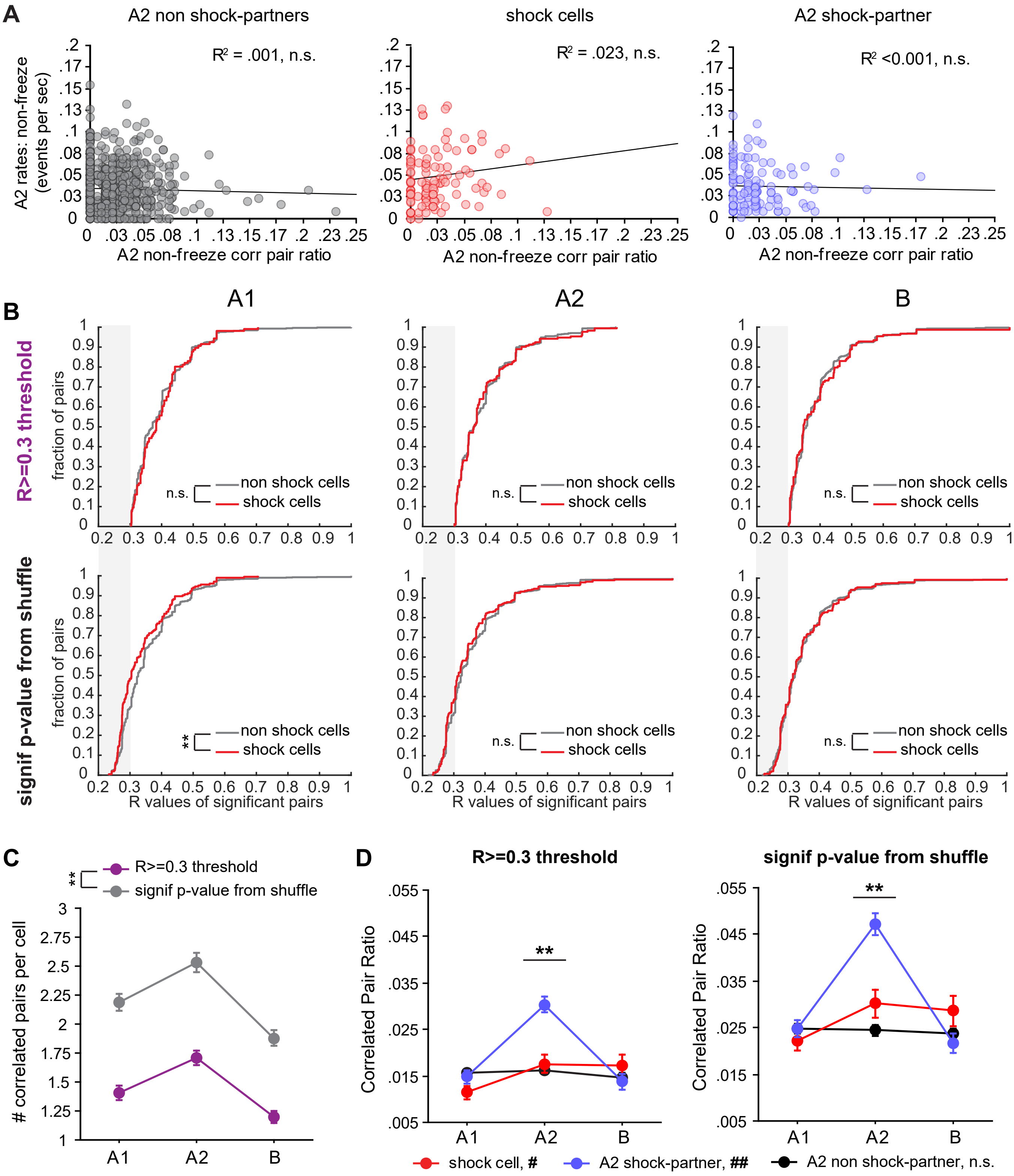

Supplementary Figure 6. Increased vCA1 correlated activity during memory retrieval is not driven by cell-type rate or R-threshold differences

a, A2 Ca^{2+} activity rate during non-freeze bouts was not correlated with correlated pair ratio across vCA1 subpopulations (N_{shock}=111, $N_{A2\ shock\text{-}partner}$=121, $N_{A2\ non\ shock\text{-}partner}$=616; Linear regression; A2 non shock-partner: $F_{(1,614)}$=0.37, p=0.54, R^2=0.001; A2 shock cell: $F_{(1,109)}$=2.53, p=0.11, R^2=0.023; A2 shock-partner: $F_{(1,119)}$=0.06, p=0.81, R^2<0.001). **b,** Cell-type specific comparison of Pearson's R thresholds as defined by R>=0.3 threshold (top) versus significant p-value relative to a shuffled R distribution (bottom); A distribution of shuffled R values was generated for each cell pair by shuffling the timing of cell events and calculating a "shuffled R" for each shuffle iteration (1000 iterations). Significant pairs were then defined by having a true R value that was significantly different from the shuffled distribution (p-value from shuffle with bonferroni correction); Top: Distribution of shock cell and non-shock cell significantly correlated pair

Supplementary Figure 6. Increased vCA1 correlated activity during memory retrieval is not driven by cell-type rate or R-threshold differences (continued)

R values as defined by R>=0.3 threshold across context conditioning days (no significant difference between cell populations across days) (KStest non-shock, shock A1: KS stat=0.082 p=0.52, A2: KS stat=0.072 p=0.40, B: KS stat=0.067 p=0.59); Bottom: Distribution of shock cell and non-shock cell significantly correlated pair R values as defined by significant p-value from shuffle across context conditioning days (KStest non-shock, shock A1: KS stat=0.149 **p=0.0005, A2: KS stat=0.075 p=0.12, B: KS stat=0.042 p=0.86); Gray boxes highlight the difference in R value range between the distributions in top and bottom panels **c,** The number of correlated pairs per cell is significantly higher across all context conditioning days when defined by p-value from shuffle distribution relative to R>=0.3 threshold (N_{cells}=848; Mann-Whitney with bonferroni corrected alpha; A1: U=272978.00 **p<0.0001, A2: U=285193.50 **p<0.0001, B: U=275487.50 **p<0.0001). **d,** The same cell-type specific effects across context conditioning days are generated when using R>=0.03 threshold (left) and significant p-value from shuffle R threshold (right) (R>=0.3 threshold; N_{shock}=111, $N_{A2\ shock\text{-}partner}$=121, $N_{A2\ non\ shock\text{-}partner}$=616; Kruskal-Wallis between groups with bonferroni alpha correction corr pair ratio: A2-A1 **p<0.0001 H(2)=49.65, B-A1 p=0.08 H(2)=5.01; wilcoxon sign rank A1,A2 corr pair ratio: shock Z=-2.47 #p<0.05, A2 shock-partner Z=-6.58 ##p<0.01, A2 non shock-partner Z=-0.61 p=0.54 | significant p-value R threshold; N_{shock}=111, $N_{A2\ shock\text{-}partner}$=162, $N_{A2\ non\ shock\text{-}partner}$=575; Kruskal-Wallis between groups with bonferroni alpha correction corr pair ratio: A2-A1 **p<0.0001 H(2)=59.33, B-A1 p=0.22 H(2)=3.00; wilcoxon sign rank A1,A2 corr pair ratio: shock Z=-2.40 #p=0.016, A2 shock-partner Z=-7.39 ##p<0.0001, A2 non shock-partner Z=-0.77 p=0.44).

Error bars, +/- s.e.m. Statistical tests comparing distributions were two-sided. Source data are provided as a Source Data file.

Supplementary Figure 7. Shock treatment does not elicit vCA1 correlated activity without context retrieval, and shock cells do not respond to tones

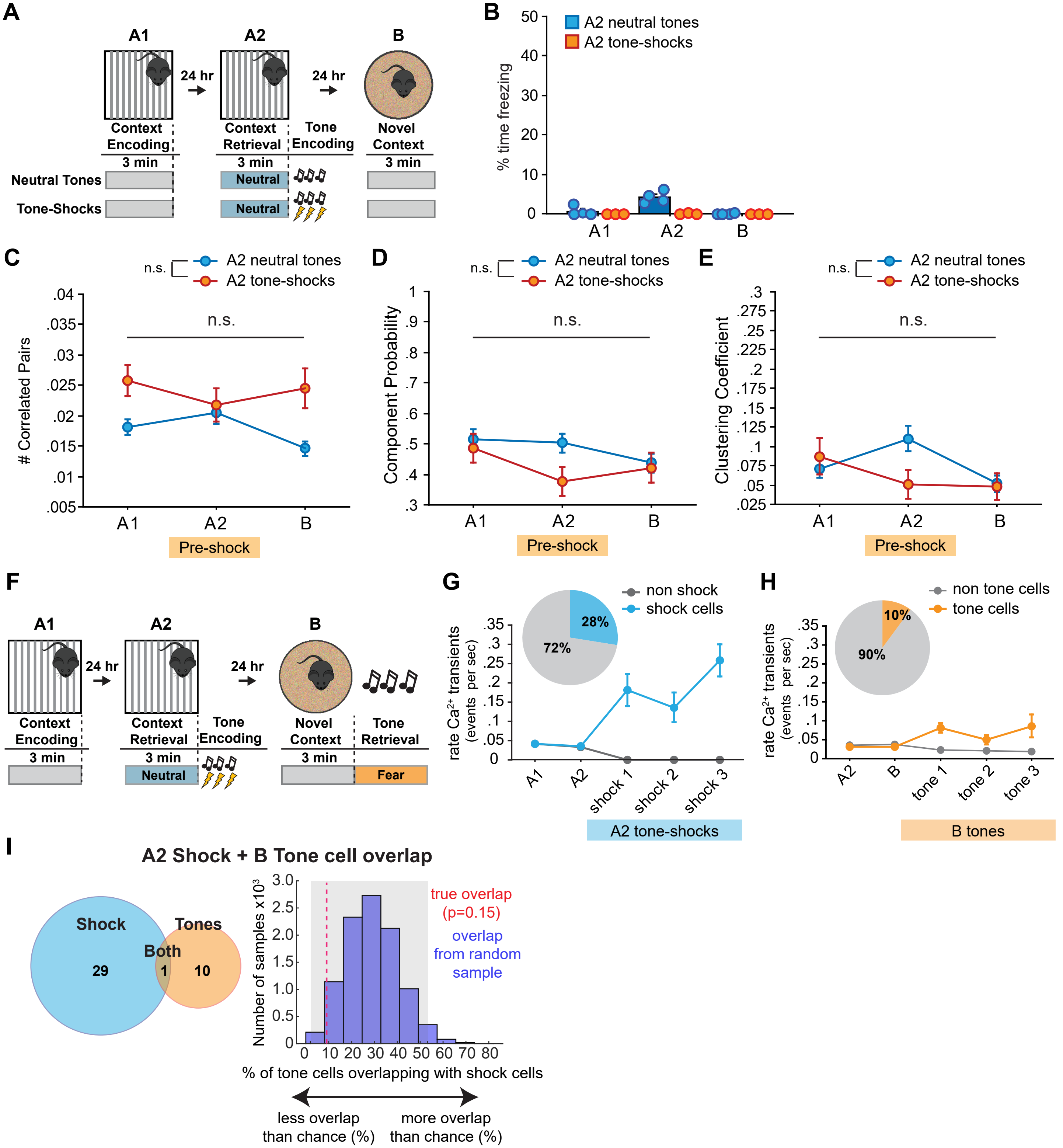

Supplementary Figure 7. Shock treatment does not elicit vCA1 correlated activity without context retrieval, and shock cells do not respond to tones

a, Experimental design, vCA1 was imaged while mice explored neutral contexts A1, A2, and B. After 3 minutes in context A2, mice were exposed to three 20-second tones either unpaired (top, Neutral Tones) or paired with a 2-sec shock (bottom, Tone-Shocks). **b,** Freezing behavior across context conditioning days (% Time freezing A2 Neutral Tone mice N=4, A2 Tone-Shock mice N=3). **c-e,** Correlation graph parameters are similar across context conditioning days between mice that received tone-shocks in A2 (orange lines), and those that never received shock treatment (neutral tones, blue lines) (Mann-Whitney between groups with bonferroni corrected alpha; $N_{neutral}$=258, $N_{tone\text{-}shock}$=109 | corr pair ratio (left); A1: U=12025.00 p=0.03, A2: U=13883.00 p=0.85, B: U=12390.50 p=0.07 | comp memb (middle); A1: U=13649.50 p=0.66, A2: U=12265.00 p=0.05, B: U=13836.50 p=0.81 | CC (right); A1: U=13742.00 p=0.73, A2: U=12404.50 p=0.07, B: U=14044.00 p=0.98). **f,** Experimental design, vCA1 was imaged while mice explored neutral contexts A1, A2, and B. After 3

Supplementary Figure 7. Shock treatment does not elicit vCA1 correlated activity without context retrieval, and shock cells do not respond to tones (continued)

minutes in context A1, mice were exposed to three 20-second tones that were paired with a 2 second shock. After 3 minutes in neutral context B, mice were exposed to the same three tones again. **g,** A subpopulation of vCA1 cells were significantly active to A2 tone-shocks (pie chart, 28% of cells were shock-responsive); Rate of Ca^{2+} transients/sec in A1, A2, and during A2 tone-shocks between shock and non-shock cells (N_{shock}=30, $N_{nonshock}$=79). **h,** A subpopulation of vCA1 cells were significantly active to B tones (pie chart, 10% of cells were tone-responsive); Rate of Ca^{2+} transients/sec in A2, B, and during B tones between tone and non-tone cells (N_{tone}=11, $N_{nontone}$=98). **i,** Tone-shock cells overlapped with tone cells at chance levels ($N_{tone\text{-}shock}$=30, N_{tones}=11; random sample overlap control analyses, 2SD range of cell overlap from mock distribution upper= 53.23% lower=1.97%; true overlap=9.1% Z=-1.44, p=0.15).

Error bars, +/- s.e.m. Statistical tests comparing distributions were two-sided. Source data are provided as a Source Data file.

Supplementary Figure 8. vCA1 shock cell correlation graphs during context conditioning

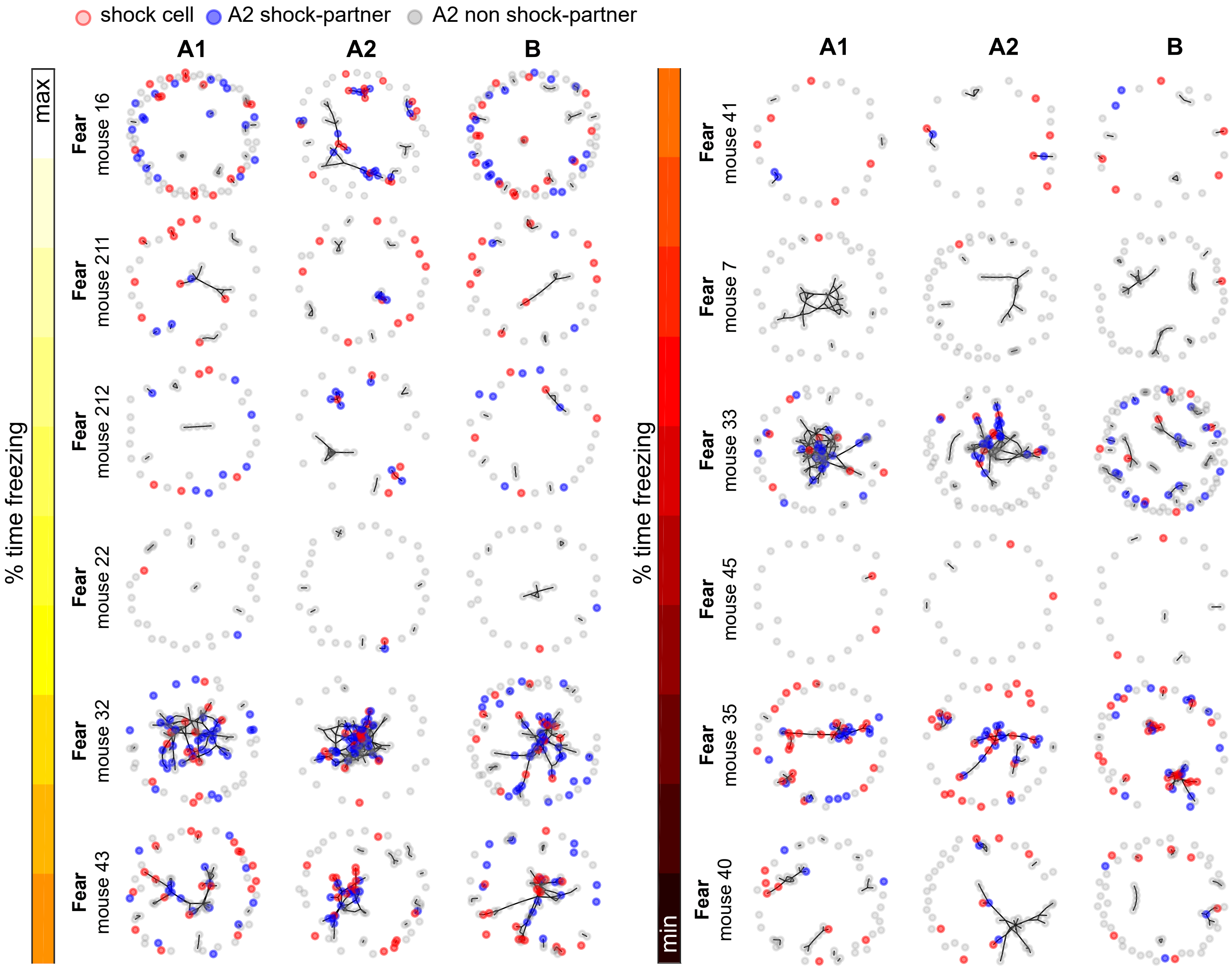

Supplementary Figure 8. vCA1 shock cell correlation graphs during fear conditioning
vCA1 correlation graphs across context conditioning days were plotted for all 12 Fear mice with shock cells labeled in red and A2 shock-partners labeled blue, demonstrating greater participation of shock cells and A2 shock-partners within correlation networks in context retrieval relative to novel context exposure A1 and B (mice sorted by rank of % time freezing starting from top left mouse 16 to bottom right mouse 40).

Supplementary Figure 9. Disruption of vCA1 shock activity during context encoding impairs correlated activity during retrieval

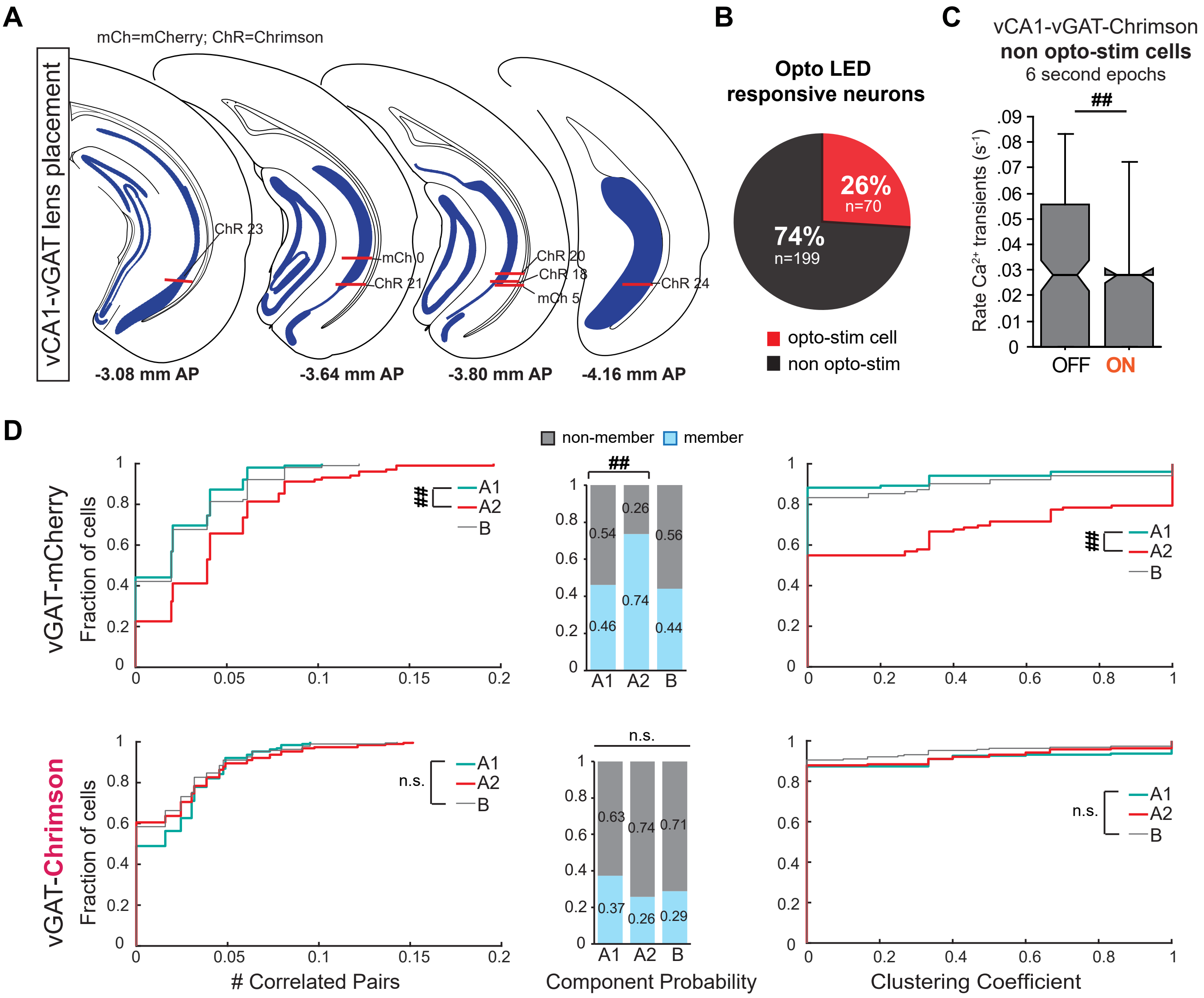

Supplementary Figure 9. Disruption of vCA1 shock activity during context encoding impairs correlated activity during retrieval
a, GRIN lens placement in vCA1 vGAT-mCherry and Chrimson imaging mice. **b,** % of vCA1 opto-stim cells in vGAT-Chrimson during homecage optoLED ON pulses (opto-stim cells defined by optoLED ON rate >1SD than a bootstrapped shuffle distribution of cell Ca^{2+} rates, $N_{opto\text{-}stim}$=70, $N_{non\ opto\text{-}stim}$=199). **c,** Non opto-stim cells have a significant decrease in Ca^{2+} rate during the first 6 seconds of optoLED ON pulses relative to the 6 secs of OFF epochs prior to light ON (wilcoxon sign rank N_{cells}=199, Z=-4.99, ##p<0.0001). **d,** Distribution of vGAT-mCherry (top row) and vGAT-Chrimson (bottom row) correlation graph parameters across context conditioning days; vGAT-mCherry but not vGAT-Chrimson mice, have a significant increase in the # of correlated pairs, component probability, and clustering coefficient during context retrieval (mCherry N_{cells}=102, Chrimson N_{cells}=190; Friedman across 3 days with post-hoc wilcoxon sign rank A1:A2 and A1:B (with bonferroni corrected alpha)| mCherry; corr pair ratio: Friedman $X^2(2)$=18.63 p<0.0001, A1:A2 Z=-4.47 ##p<0.0001, A1:B Z=-1.20 p=0.23; comp memb: Friedman $X^2(2)$=12.41 p=0.0020, A1:A2 Z=-3.26 ##p<0.0011, A1:B Z=-0.25 p=0.80; CC: Friedman $X^2(2)$=17.24 p=0.0002, A1:A2 Z=-4.65 ##p<0.0001, A1:B Z=-0.87 p=0.38 | Chrimson; corr pair ratio: Friedman $X^2(2)$=3.99 p=0.14, comp memb: Friedman $X^2(2)$=3.06 p=0.22, CC: Friedman $X^2(2)$=0.22 p=0.90).
Error bars, +/- s.e.m. Box plots, min/max, interquartile range, median. Statistical tests comparing distributions were two-sided. Source data are provided as a Source Data file.

Supplementary Figure 10. vCA1 shock period silencing disrupts correlated activity during memory retrieval

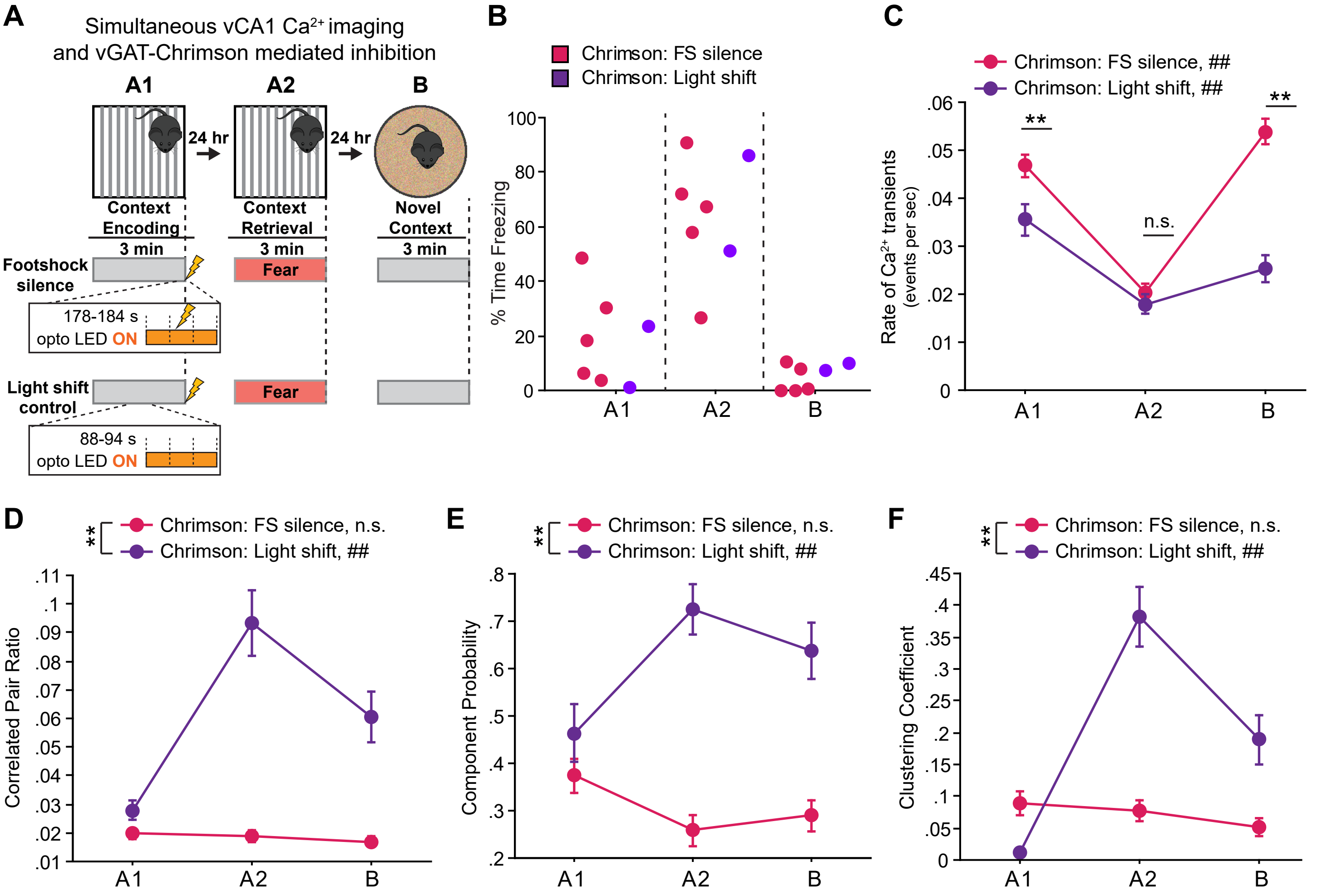

Supplementary Figure 10. vCA1 shock period silencing disrupts correlated activity during memory retrieval
a, Experimental design, vGAT-Chrimson mice were imaged in a 3-day contextual fear paradigm, with the optoLED turned ON either during the footshock in A1 to selectively disrupt the vCA1 shock response during context encoding (Footshock silence mice, opto LED ON from 178-184 seconds in A1) or during a Light shifted control period outside of the footshock (Light shift control mice, opto LED ON from 88-94 seconds in A1). **b,** Freezing behavior across context conditioning days after 6 seconds of silencing in A1 during the footshock or a light shift period ($N_{FS\text{-}silence}$=5, $N_{Light\text{-}shift}$=2). **c,** vCA1-mCherry and vCA1-Chrimson neurons exhibited a significant decrease in Ca^{2+} event rate during context retrieval (A2) (Chrimson N_{cells}=190, Light-shift N_{cells}=69; Mann-Whitney between groups with bonferroni corrected alpha: A1 U=5145.50 *p=0.01, A2 U=6492.00 p=0.91, B U=3302.50 **p<0.0001| wilcoxon sign rank with bonferroni corrected alpha; Chrimson: A1:A2 Z=-9.13 ##p<0.0001; Light-shift: A1:A2 Z=-5.05 ##p<0.0001). **d-f,** Disrupting the vCA1 shock response during context encoding impaired the formation of increased correlated activity during context retrieval across all correlation graph parameters in vGAT-Chrimson mice, but not in vGAT-Chrimson Light shift control mice (Chrimson N_{cells}=190, mCherry N_{cells}=69; Mann-Whitney between groups with bonferroni corrected alpha; corr pair ratio A2-A1 U=3808.50 **p<0.0001, B-A1 U=4944.00 **p=0.0025; comp probability A2-A1 U=4611.00 **p=0.0003, B-A1 U=5193.00 **p=0.0106; CC A2-A1 U=3173.00 **p<0.0001, B-A1 U=4844.00 **p=0.0013). Error bars, +/- s.e.m. Statistical tests comparing distributions were two-sided. Source data are provided as a Source Data file.

Supplementary Figure 11. vCA1 context-shock silencing in vGAT-Chrimson mice does not disrupt correlated activity formation in tone-shock conditioning

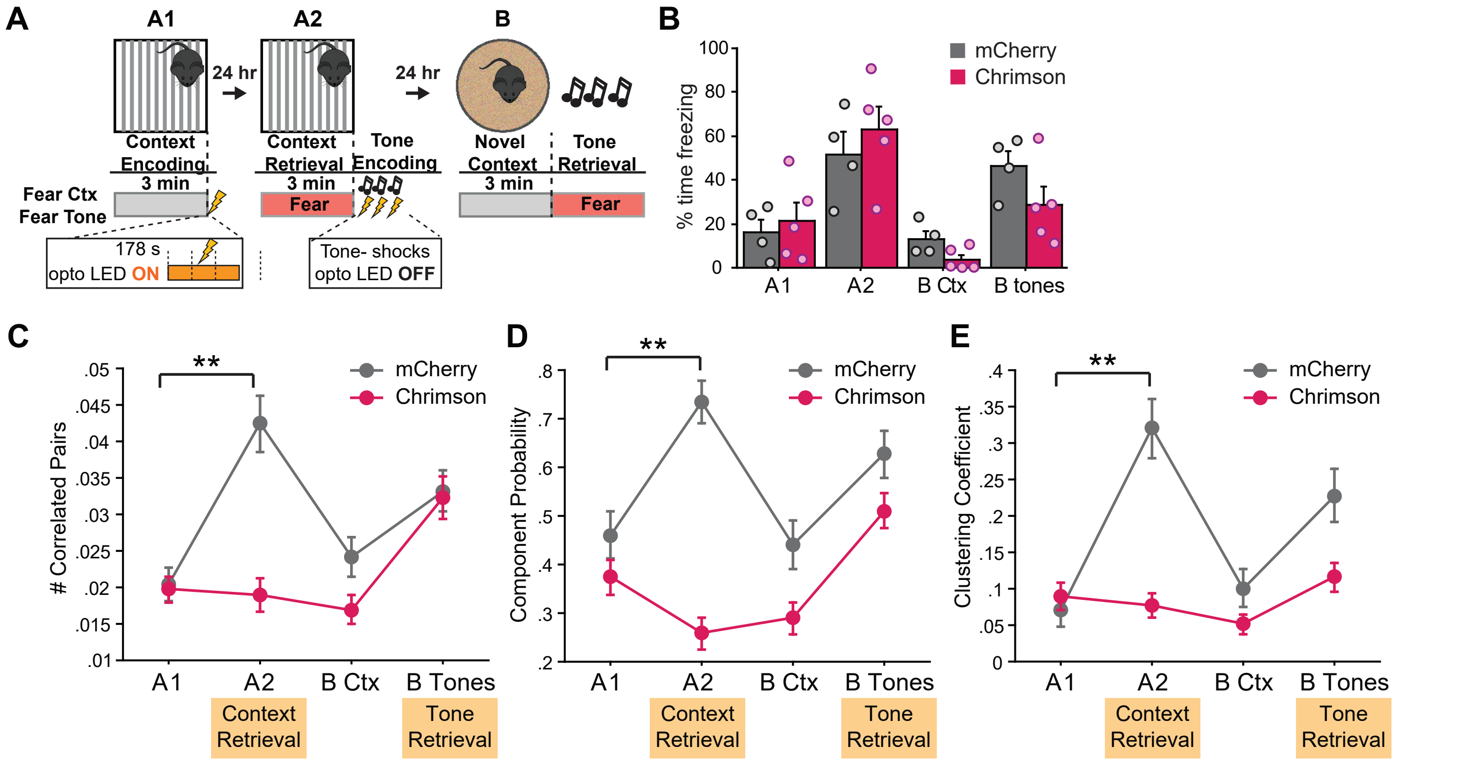

Supplementary Figure 11. vCA1 context-shock silencing in vGAT-Chrimson mice does not disrupt correlated activity formation in tone-shock conditioning

a, Experimental design: context-shock opto ON and tone-shock opto OFF; vGAT-Chrimson and mCherry mice were silenced during context shock conditioning (6 seconds of silencing in A1), but the following day after context retrieval in A2, mice received 3 tone-shock pairings under normal conditions (with no opto silencing). Tones were presented to mice after 3 minutes of context B exploration in day 3 to test tone retrieval. **b,** vCA1-mCherry and Chrimson mice froze similar amounts during normal tone retrieval (B tones) after unilateral vCA1 context-shock silencing in A1 (repeated-measures ANOVA; % time freezing*genotype interaction $F_{(1,7)}=2.27$, $p=0.11$, $N_{mCherry}=4$, $N_{Chrimson}=5$). **c-e,** While context-shock inhibition disrupted vCA1 correlated activity in vGAT-Chrimson mice (but not vGAT-mCherry) during context retrieval, tone-shock training in optoLED OFF conditions elicited similar amounts of correlated activity during tone retrieval (Btones-BCtx) between groups (mCherry $N_{cells}=102$, Chrimson $N_{cells}=190$; Mann-Whitney between groups with bonferroni corrected alpha: corr pair ratio A2-A1 U=6713.00 **p<0.0001, BTones-BCtx U=9324.50, p=0.60, comp probability A2-A1 U=6776.00 **p<0.0001, BTones-BCtx U=9563.50, p=0.85 ; CC A2-A1 U=6376.00 **p<0.0001, BTones-BCtx U=8825.00, p=0.21).
Error bars, +/- s.e.m. Statistical tests comparing distributions were two-sided. Source data are provided as a Source Data file.
