# Supplementary material for: Contextual fear memory retrieval by correlated ensembles of ventral CA1 neurons
Source: Nat Commun. 2020 Jul 13;11:3492. doi: 10.1038/s41467-020-17270-w (PMC7359370; doi:10.1038/s41467-020-17270-w)
Supplement: Supplementary file 3 — Reporting Summary [file 41467_2020_17270_MOESM3_ESM.pdf]

nature research

Corresponding author(s): Rene Hen

Last updated by author(s): 6/8/2020

Reporting Summary

Nature Research wishes to improve the reproducibility of the work that we publish. This form provides structure for consistency and transparency in reporting. For further information on Nature Research policies, see Authors & Referees and the Editorial Policy Checklist .

Statistics

For all statistical analyses, confirm that the following items are present in the figure legend, table legend, main text, or Methods section.

n/a	Confirmed	
☐	☒	The exact sample size (*n*) for each experimental group/condition, given as a discrete number and unit of measurement
☐	☒	A statement on whether measurements were taken from distinct samples or whether the same sample was measured repeatedly
☐	☒	The statistical test(s) used AND whether they are one- or two-sided<br>*Only common tests should be described solely by name; describe more complex techniques in the Methods section.*
☒	☐	A description of all covariates tested
☐	☒	A description of any assumptions or corrections, such as tests of normality and adjustment for multiple comparisons
☐	☒	A full description of the statistical parameters including central tendency (e.g. means) or other basic estimates (e.g. regression coefficient) AND variation (e.g. standard deviation) or associated estimates of uncertainty (e.g. confidence intervals)
☐	☒	For null hypothesis testing, the test statistic (e.g. *F*, *t*, *r*) with confidence intervals, effect sizes, degrees of freedom and *P* value noted<br>*Give P values as exact values whenever suitable.*
☒	☐	For Bayesian analysis, information on the choice of priors and Markov chain Monte Carlo settings
☐	☒	For hierarchical and complex designs, identification of the appropriate level for tests and full reporting of outcomes
☐	☒	Estimates of effect sizes (e.g. Cohen's *d*, Pearson's *r*), indicating how they were calculated

Our web collection on statistics for biologists contains articles on many of the points above.

Software and code

Policy information about availability of computer code

Data collection	Fear conditioning behavior was conducted in a commercially available shock box (Med Associates, ENV-010MD), synchronized with behavioral video recording with EthovisionXT 11 video software (Noldus, Leesburg, VA), and Inscopix miniaturized microscope acquisition software (Inscopix, Palo Alto, CA).
Data analysis	Freezing behavior was scored using ObserverXT 12 software (Noldus, Leesburg, VA). Calcium video image processing was performed using commercially available Mosaic and Inscopix Data Processing software (versions 1.0.5b and 1.2.0, Inscopix, Palo Alto, CA). Cell segmentation was performed using the open-source algorithm Constrained Non-negative Matrix Factorization for microEndoscopic data (CNMF-E). Calcium transient events were defined with a custom Matlab event detection algorithm and ran on the de-noised calcium trace extracted from CNMF-E. Correlation graphs were constructed using the NetworkX Python package, and all subsequent analyses were conducted with custom Matlab and Python codes, available at the following public repository: https://github.com/jaberry/Jimenez_2020

For manuscripts utilizing custom algorithms or software that are central to the research but not yet described in published literature, software must be made available to editors/reviewers. We strongly encourage code deposition in a community repository (e.g. GitHub). See the Nature Research guidelines for submitting code & software for further information.

Data

Policy information about availability of data

All manuscripts must include a data availability statement. This statement should provide the following information, where applicable:

- Accession codes, unique identifiers, or web links for publicly available datasets
- A list of figures that have associated raw data
- A description of any restrictions on data availability

The datasets generated during and/or analyzed during the current study are available at the following public repository (https://github.com/jaberry/Jimenez_2020) and from the corresponding author on reasonable request.

Field-specific reporting

Please select the one below that is the best fit for your research. If you are not sure, read the appropriate sections before making your selection.

☒ Life sciences ☐ Behavioural & social sciences ☐ Ecological, evolutionary & environmental sciences

For a reference copy of the document with all sections, see nature.com/documents/nr-reporting-summary-flat.pdf

Life sciences study design

All studies must disclose on these points even when the disclosure is negative.

Sample size	For all behavioral experiments, the number of mice was determined based on previous publications that utilized optogenetics to manipulate hippocampal activity during contextual fear conditioning (Kheirbek et al. 2013, Lovett-Barron et al. 2014, Jimenez et al. 2018). For imaging experiments, the number of mice was determined based on previous publications that utilized freely-moving calcium imaging (Ziv et al., 2013; Jennings et al., 2015, Jimenez et al. 2018, Anacker et al., 2018).
Data exclusions	For all correlated activity analysis, only mice with >=20 cells detected per FOV were included in the final analysis given the sampling limitation of potential correlated pair partners in sparse FOVs. This exclusion criteria was pre-established.
Replication	All replication attempts were successful. The effect of increased correlated activity in vCA1 was replicated in 5 independent datasets, and in different strains of mice (C57 and vGAT-Cre). The effect of increased shock cell proportion in the vCA1-BA projection was consistent across experimental mice analyzed. The effect of disrupted correlated activity during memory retrieval following vCA1 silencing during encoding was replicated in an independent dataset within the lab, and is consistent across animals included in the manuscript. The effect of disrupted time freezing during memory retrieval following bilateral vCA1 silencing during the shock period in contextual memory encoding was replicated in an independent dataset within the lab.
Randomization	Mice were randomly assigned to experimental groups prior to surgery, and datasets for experimental groups were collected in parallel.
Blinding	All data collection was ran by automated software, with the same software settings used across all animal subjects. Investigators were blinded to animal group assignment at time of behavioral analysis (freeze scoring), and all other data processing was ran by automated software packages.

Reporting for specific materials, systems and methods

We require information from authors about some types of materials, experimental systems and methods used in many studies. Here, indicate whether each material, system or method listed is relevant to your study. If you are not sure if a list item applies to your research, read the appropriate section before selecting a response.

Materials & experimental systems

n/a	Involved in the study	
☒	☐	Antibodies
☒	☐	Eukaryotic cell lines
☒	☐	Palaeontology
☐	☒	Animals and other organisms
☒	☐	Human research participants
☒	☐	Clinical data

Methods

n/a	Involved in the study	
☒	☐	ChIP-seq
☒	☐	Flow cytometry
☒	☐	MRI-based neuroimaging

Animals and other organisms

Policy information about studies involving animals; ARRIVE guidelines recommended for reporting animal research

Laboratory animals	Adult male C57BL/6J mice were supplied by the Jackson Laboratory and used for experiments, and vGAT-IRES-Cre mice were bred on a C57BL/6J background and both male and female mice were used for experiments and balanced between groups. All mice were used for experiments starting at 8 weeks of age. All mice were housed 2-5 per cage on a 12 hour light/dark schedule with lights off at 6:00 PM, with ambient temperature ~74 degrees Fahrenheit and ~20% humidity. Ca2+ imaging mice were single housed with an enrichment hut immediately following surgery to avoid damage to their imaging hardware.
Wild animals	No wild animals were used in the study.
Field-collected samples	No field collected samples were used in the study.
Ethics oversight	Procedures were conducted in accordance with the U.S. NIH Guide for the Care and Use of Laboratory Animals and the New York State Psychiatric Institute Institutional Animal Care and Use Committees at Columbia University.

Note that full information on the approval of the study protocol must also be provided in the manuscript.
